# Moderate Changes in the Circadian System of Alzheimer's Disease Patients Detected in Their Home Environment

**DOI:** 10.1371/journal.pone.0146200

**Published:** 2016-01-04

**Authors:** Kamila Weissová, Aleš Bartoš, Martin Sládek, Marta Nováková, Alena Sumová

**Affiliations:** 1 Department of Neurohumoral Regulations, Institute of Physiology, the Czech Academy of Sciences, Prague, Czech Republic; 2 Charles University in Prague, Third Faculty of Medicine, Department of Neurology, Prague, Czech Republic; 3 National Institute of Mental Health, Klecany, Czech Republic; McGill University, CANADA

## Abstract

Alzheimer's disease (AD) is a neurodegenerative disease often accompanied with disruption of sleep-wake cycle. The sleep-wake cycle is controlled by mechanisms involving internal timekeeping (circadian) regulation. The aim of our present pilot study was to assess the circadian system in patients with mild form of AD in their home environment. In the study, 13 elderly AD patients and 13 age-matched healthy control subjects (the patient's spouses) were enrolled. Sleep was recorded for 21 days by sleep diaries in all participants and checked by actigraphy in 4 of the AD patient/control couples. The samples of saliva and buccal mucosa were collected every 4 hours during the same 24 h-interval to detect melatonin and clock gene (*PER1* and *BMAL1*) mRNA levels, respectively. The AD patients exhibited significantly longer inactivity interval during the 24 h and significantly higher number of daytime naps than controls. Daily profiles of melatonin levels exhibited circadian rhythms in both groups. Compared with controls, decline in amplitude of the melatonin rhythm in AD patients was not significant, however, in AD patients more melatonin profiles were dampened or had atypical waveforms. The clock genes *PER1* and *BMAL1* were expressed rhythmically with high amplitudes in both groups and no significant differences in phases between both groups were detected. Our results suggest moderate differences in functional state of the circadian system in patients with mild form of AD compared with healthy controls which are present in conditions of their home dwelling.

## Introduction

Alzheimer's disease (AD) is a neurodegenerative disorder causing a variety of irreversible cognitive impairments leading to dementia. Apart from memory deficits [1], AD pathological symptoms involve impairments in regulation of various physiological processes, including circadian regulations of behavior, sleep patterns and hormonal secretion [2]. These physiological functions are temporally controlled by a circadian system which consists of the central clock in the suprachiasmatic nuclei (SCN) and peripheral clocks in neuronal and non-neuronal cells and tissues [3–5]. The central SCN clock drives systemic rhythms, mainly sleep/wake cycle and rhythm in pineal hormone melatonin levels [6], and synchronizes the peripheral clocks which drive rhythmically the tissue specific physiological programs [3]. The circadian signal is generated at the cellular level via autonomous molecular mechanism which drives rhythmically expression of clock genes, namely *PER1*,*2*, *CRY1*,*2*, *REV-ERBα*, and *BMAL1*. As a result of the molecular clock mechanism, circadian expression of *PER1*,*2*, *CRY1*,*2*, and *REV-ERBα* is in anti-phase to that of *BMAL1* (reviewed in [7]).

Among people over 65 years old, more than 80% suffer from abnormalities in sleep/wake rhythmicity [8–10]. The function of the circadian system changes with age with an individually variable progression speed in elderly people even without AD pathology [11]. Therefore, it might be difficult to distinguish between the age- and AD-related modifications in circadian regulation These age-related changes of the circadian system involve alterations in amplitudes and phases of circadian rhythms [12], as well as changes in timing of the sleep/wake cycle with respect to the circadian cycle, i.e., shortening the phase angle of entrainment [13, 14]. In AD patients, incidence of sleep/wake cycle disturbances was found to be higher compared to age-matched controls. They mostly exhibit exacerbated disruption of sleep, such as fragmented nighttime sleep and a higher frequency and duration of nighttime awakenings and daytime sleep episodes (naps) [9, 15, 16]. Importantly, disruption of the sleep/wake cycle was diagnosed in AD patients already in mild and moderate stages of the disease [17].

Due to the SCN control, production of hormone melatonin exhibits pronounced circadian rhythms so that its levels are high during the subjective night and very low during the subjective day [18]. In healthy elderly people, the circadian rhythm in melatonin levels was dampened because their nocturnal melatonin secretion was decreased [19]. However, this issue is rather controversial because other studies did not confirm that reduction of plasma melatonin concentration is a general characteristic of healthy aging [20, 21]. In AD patients, more pronounced decrease in melatonin secretion was detected at early stages of the disease when their cognitive functions were still intact [22] and the rhythm dampening correlated with the AD neuropathology progression [22, 23]. These results suggest that in AD patients, the circadian function of the central SCN clock which drives rhythms in the aforementioned functions, might deteriorate further beyond that of what happens in elderly without the AD pathology [24].

It is still not known whether the worsening of the circadian regulation in AD is due to changes of the SCN morphology and function or due to changes of functions downstream the central clock. The age-dependent changes in the SCN were detected in healthy elderly but they occurred earlier and were more pronounced in AD patients [25–32]. However, whether the morphologic pathology causally accounts for the circadian SCN dysfunction is not clear. It is possible that the SCN clock mechanism itself is not affected in AD patients but the nuclei are disconnected from the rest of the brain. Such disconnection would result in aberrant circadian regulation of the downstream brain areas which contain subordinate circadian clocks. For example, in healthy subjects, clock gene expression was found to be rhythmic in the bed nucleus of stria terminalis (BNST), the cingulate cortex and the pineal gland [33, 34]. In the brains of AD patients, most of these clocks also exhibited well pronounced 24-hour rhythmicity, however, their mutual synchronization was altered compared to controls [35]. In contrast, the rhythms in clock gene expression in pineal glands were completely lost in both clinical and preclinical AD patients [33].

As mentioned above, the aberrant circadian regulation was shown to precede clinical onset of memory deficits in AD (reviewed in [36]) and might be associated with AD development [37], Therefore, the present study was aimed to find out whether disruption of the circadian system, more pronounced than that caused by physiological aging, is present in patients with the mild form of AD. Specifically, the study was designed to reveal whether the circadian disruption is present during the everyday life of patients who do not require hospitalization and live in their home environment. To achieve this, the couples of age-matched patients and their healthy spouses (who lived together and took care of them) were examined. The functional state of the circadian system was assessed based on the analysis of daily profiles of behavioral patterns, melatonin levels in saliva and clock gene expression in the peripheral cells of buccal mucosa.

## Materials and Methods

### Participants

In the study, 13 healthy subjects (6 females and 7 males) and 13 AD patients (7 females and 6 males) were enrolled. The subjects of both groups were matched for age (mean ± S.E.M.; controls: 78.1 ± 2.0 years, AD patients: 78.9 ± 1.9 years) and education level (mean ± S.E.M. of years of schooling; controls: 14.5 ± 2.0, AD patients: 14.0 ± 3.0). The exclusion criteria for participation in the study were traveling across the time zones or working night shifts one month or less before the beginning of the study. For inclusion of patients in the study, the willingness of their spouses living in the same home environment to cooperate in the study was conditional.

The AD patients were recruited from the outpatients of the Memory Clinic, AD Center, Charles University in Prague, Czech Republic. The diagnosis was based on diagnostic guidelines using clinical evaluation in combination with results of brain magnetic resonance imaging (hippocampal atrophy), single photon emission computed tomography (SPECT, temporo-parietal hypoperfusion) or analysis of cerebrospinal fluid concentrations (abnormal for amyloid-beta, total tau protein or phosphorylated tau protein) ([38]). They had dementia due to the AD; their cognition, examined with the MMSE, was significantly impaired when compared to the controls (21 ± 3 and 29 ± 1 points, respectively). All AD patients were on stable treatment of antidementia drugs (acetylcholinesterase inhibitors, memantine). Prior to and during the sampling period, the AD patients were free of sleep medication; only one subject reported previous using of sleep medication which was withdrawn during the testing period. The clinical and biomarker characteristics of 13 patients with Alzheimer disease involved in the study are summarized in S1 Table.

The controls included the elderly patient's spouses of similar age and gender proportion as the AD patients group. They were exposed to the same photic and non-photic environmental cues of their home dwellings long before and throughout the study. The spouses had to be without diagnosis of AD or other form of dementia according to clinical judgment based on long-term observations during visits with their patients prior to this study. They had normal cognitive functions assessed with the MMSE (Mini-Mental State Examination) [39] and the clock drawing test. They reported good health, were free of medication before and throughout the study and had no sleep problems; only one control subject reported previous use of a sleep drug which was withdrawn during the testing period. The controls helped patients with sampling and checked that it was performed correctly (especially important for collection of buccal samples) and at proper time intervals.

Most subjects of both groups had age-adequate deterioration in eye function, they wore glasses (10 controls and 13 AD patients) and some of them reported cataract (8 controls and 7 AD patients).

### Ethical statement

The patients and control subjects were informed in detail about the purpose and procedures of the study and signed an informed consent form. The protocol and the consent form were in agreement with the Declaration of Helsinki and were approved by the Ethics Committees of the Institute of Physiology, the Czech Academy of Sciences and of the University Hospital Kralovske Vinohrady Prague Psychiatric Centre, Alzheimer Disease Centre, Czech Republic.

### Protocol of the study and sample collection

**Fig 1:**
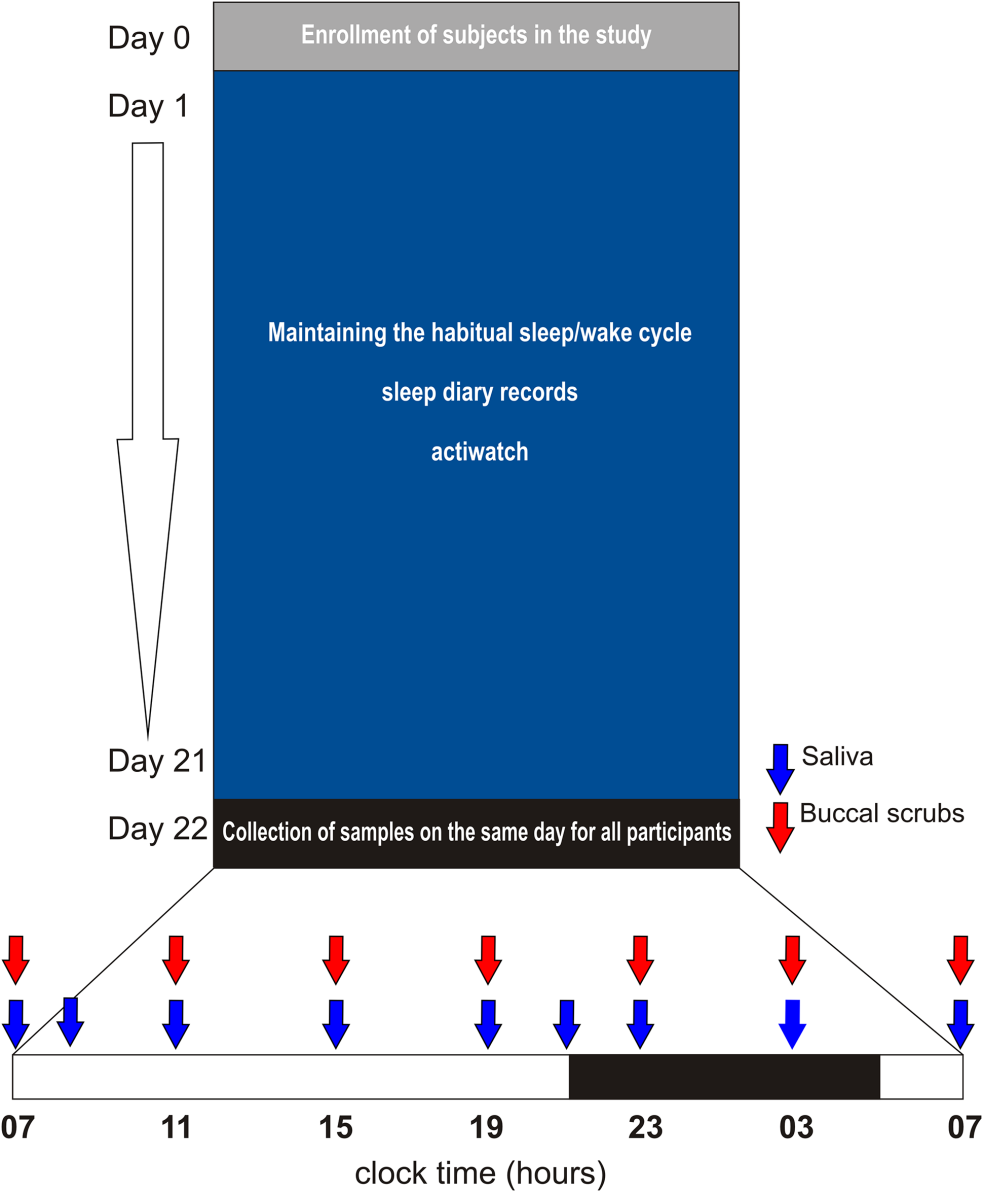
Protocol of the study. The study began (day 1) with recording of sleep/wake schedule in diaries (all participants) together with Actiwatch monitoring (4 couples) for 21 days. During the monitored period, the subjects maintained their habitual sleep/wake regime. Thereafter, the samples were collected throughout the 24 h on the same day from all subjects. Time when saliva (blue arrows) and buccal mucosa samples (red arrows) were collected is depicted. The dark bar on the time scale corresponds to hours between the sunset and the sunrise on the sampling day. For more details, see Materials and Methods.

The protocol of the study is summarized in Fig 1. Before beginning of the study, the participants were informed about the basic scientific background and the experimental design of the study at an instructional meeting. Especially, importance of light intensity control on the sampling day was explained. To check whether the instructions were understood and feasible to follow, the authors (KW) visited the subjects' homes to help and/or advice them with adjustments in order to ensure that the light intensity during the night when the sampling was performed did not exceed the limit (see below). The controls and AD patients were asked to maintain their normal sleep schedule throughout the entire study. All subjects were examined during the same period of April—May 2013.

The study started with sleep/wake cycle recording using individual sleep diaries in all study participants (the diaries of AD patients were recorded by their spouses) and Actiwatches in 4 randomly selected couples for 21 days prior to sampling. Thereafter, collection of samples from all study participants was accomplished within one day (the same day for all participants) in the patient/control couple's homes. This arrangement ensured exactly the same outside seasonal and lighting conditions during the study for all subjects. On the day of sampling, the sunset occurred at 21:02 and the sunrise at 4:57. The ceiling lights were turned off during the habitual sleeping time and turned on during the habitual waking time. The light exposure during the night was controlled and was the same for the controls and patients; dim light was provided by the bedside lamp covered with a fabric and was controlled not to exceed 20 lux (measured by luxmeter at the time of the researcher visit). Light of this intensity did not to affect melatonin levels in our previous studies [40–43]. During the entire 24-h interval of sampling, the subjects were not allowed to drink alcoholic and caffeinated beverages, use chewing gum and brush their teeth. Moreover, for 1 h before each sampling, they did not consume food or drink. The subjects provided saliva samples directly into test tubes that were stored in the fridge overnight. Thereafter, in the morning the samples were transferred to the laboratory and stored at −20°C until assay. Immediately after providing the saliva, buccal mucosa samples were collected by gently scratching the inner cheek on both sides using a cytological brush. The oral mucosa samples were immediately placed into RNAlater reagent (Sigma-Aldrich, St. Louis, USA) and maintained at room temperature overnight. In the morning following the sampling, they were transferred to the laboratory together with the saliva samples and stored at −20°C until analysis. The sampling of saliva and buccal swabs began at 07:00 and continued every 4 h throughout the 24 h until 07:00 on the following day; the last sampling at 07:00 was performed in darkness. Additional time points when only saliva but not buccal scrubs were collected occurred at 21:00 and 09:00 h. This protocol provided more frequent sampling of saliva around the time of expected melatonin rise and decline and, at the same time, prevented damaging the oral mucosa by too frequent brushing. The sampling procedure was tolerated by all participants without any complaints.

### Activity and sleep/wake recording

Sleep/wake schedules were recorded for all participants in sleep diaries during 21 days preceding the sampling (one couple was able to keep the record only for 17 days). The subjects were asked to log the time intervals (30 min resolution) when they had been asleep during 24 h. The records were analyzed manually to determine timing of the sleep onset and offset in order to count the sleep duration as well as to identify daytime sleep episodes, i.e., naps (see below).

The subjective recordings of sleep/wake cycle by diaries obtained from all study participants were checked in 4 randomly selected patient/control couples who wore Actiwatch devices (AW4 model; Cambridge Neurotechnology Ltd, UK) on their non-dominant hands during the 21 days. Unfortunately, the number of Actiwatches which could be used in the study, was limited to 8, and therefore, it was not possible to monitor all participants of the study. The device recorded movement in 1-min bins and the data were analyzed by Actiwatch Activity & Sleep Analysis V 5.42 software (Cambridge Neurotechnology Ltd, UK). The activity patterns were graphically presented as daily records (actigraphs) throughout the 21 days. For better clarity, the records were double plotted, i.e., each line of the actigraphs represented two consecutive days and the next line started with the record of the previous day. The data of Actiwatch analysis provided information on the overall daily activity profiles, sleep parameters and daytime naps. For the overall daily activity profiles, the results were expressed as sums of 1 min activity epochs recorded within 30-min intervals throughout the 24 h. The data for each interval were cumulated for the 21-day recording period separately for controls and AD patients and were expressed as a mean activity ± S.E.M. of each of the 30-min values. For sleep duration and sleep quality analysis, following parameters were analyzed: i) *assumed sleep duration*—i.e., the time interval between falling asleep and waking, based on the data from diary; ii) *number/duration of wake bouts*—i.e., the number and duration of episodes which the algorithm recognized as a state of wakefulness; iii) *percentage of real sleep duration*—i.e., percentage of the real sleep (without waking episodes) of the entire sleep duration; iv) *sleep efficiency*—i.e., the percentage of sleep duration of the entire time spent in the bed; v) *fragmentation index*—i.e., the percentage of 1-min intervals of immobility vs. number of all 1-min intervals during the sleep period. For the nap analysis, the software setting determined a nap as an interval of inactivity longer than 15 min during the time of day that the subject designed as "active" daytime when being awake (based on diary data). To distinguish between immobility in vigilance state, the interval marked as naps by the software were cross-checked with the records in the diaries.

### Melatonin assay

A direct double-antibody radioimmunoassay was used for the melatonin assay (Bühlmann Laboratories, Allschwil, Switzerland) as previously described (26). The kit was used according to the manufacturer’s instructions. The analytical sensitivity was 0.2 pg/ml. The intra-assay coefficient of variation was 3% for samples of 18.5 ± 1.0 pg/ml and 4% for samples of 2.4 ± 0.2 pg/ml. The inter-assay coefficient of variation was 12% for samples of 18.5 ± 1.0 pg/ml and 14% for samples of 2.4 ± 0.2 pg/ml. The melatonin levels at each time point were expressed in pg/ml as mean ± S.E.M.

### Determination of clock gene expression by quantitative real-time polymerase chain reaction (RT-qPCR)

As described previously [41], mRNA was isolated using the Dynabeads mRNA Direct Micro Kit (Invitrogen, Carlsbad, California, USA) and the whole mRNA sample was reverse-transcribed using the SuperScript VILO cDNA Synthesis Kit (Invitrogen) in 10-μl reactions incubated at 42°C for 1 h. The cDNA was then diluted 1:2 with RNase-free water and 2 μl was used to determine gene expression in 16 μl qPCR reaction. Each reaction also contained 10.3 μl of PCR-grade water, 3.1 μl of HOT FIREPol Probe qPCR Mix Plus (Solis BioDyne, Estonia) and 0.6 μl of TaqMan Gene Expression Human FAM-MGB assay (Life Technologies, CA, USA) specific for the following genes: *Period 1* (*PER1*, NM 002616, cat. no. Hs01092603_m1), *Aryl hydrocarbon receptor nuclear translocator-like* (*ARNTL*, syn. *BMAL1*, NM 001178, cat. no. Hs00154147_m1), *Glyceraldehyde-3-phosphate dehydrogenase* (*GAPDH*, NM 002046, cat. no. Hs99999905_m1) and *Ribosomal protein large P0* (*RPLP0*, NM 001002, cat. no. Hs99999902_m1). The reaction were amplified in a sealed 96-well microplate using ViiA 7 Real-Time PCR system (Life Technologies, CA, USA) with default cycling conditions for TaqMan assay (50°C—2 min; 95°C—10 min; 50x 95°C—15 sec, 60°C—1 min). Resulting amplification curves were analyzed using ViiA7 Software 1.1 (Life Technologies, CA, USA). Negative control without the cDNA showed no amplification. To generate calibration curves and compare differences between samples, a serially diluted cDNA from total RNA isolated from cultured human primary fibroblasts was used. The levels of expression of *PER1* and *BMAL1* were normalized to the mean expression of both reference genes (*GAPDH* and *RPLP0*).

### Statistical Analysis

The data of activity levels for AD patients and healthy controls were plotted as the mean ± SEM for each 30-min bin of the 24 h interval during the 21 days of recording. The profiles in both groups were compared using the repeated measures 2-way ANOVA. The parameters from the sleep quality and nap analyses were averaged for each group and compared between controls and AD patients by the Student's t-test corrected for multiple comparisons.

The data for melatonin levels at each time point were plotted as mean ± SEM for each group and compared by repeated measures 2-way ANOVA. The individual melatonin profiles were analyzed by cosinor analysis (see below) to assess the presence/absence of daily rhythms in each subject. Also, area under the curve was calculated for each individual melatonin profile, averaged for the group of healthy subjects and AD patients and the values were compared between the groups by t-test.

The data for clock gene expression were expressed as mean ± SEM for each group and analyzed by cosinor analysis (see below). The acrophases of the profiles were compared by t-test. The expression profiles of controls and AD patients were compared by repeated measures 2-way ANOVA.

Cosinor analysis: The data were fitted with two alternative regression models: either a horizontal straight line (null hypothesis) or a single cosine curve (alternative hypothesis) as defined by the equation Y = mesor+[amplitude*cos(2*π*(X-acrophase)/period)] with a constant period of 24 h. The extra sum-of-squares F test was used for comparison, and cosine curve parameters, such as amplitude (*i*.*e*., the difference between the peak or trough and the mean value of a cosine curve), acrophase (*i*.*e*., the phase angle of the peak of a cosine curve) and the coefficient of determination R^2^ (*i*.*e*., goodness of fit) were calculated unless the P value exceeded 0.05.

The statistical, information theory and least-squares regression methods implemented in Prism 6 software (GraphPad, La Jolla, USA) were applied. Where appropriate, data were checked for normal distribution.

## Results

### Total inactivity period during 24 h and number of daytime naps are increased in AD patients

For analysis of the overall rest/activity and sleep/wake daily patterns and of the nighttime and daytime sleep parameters, data from sleep diaries (13 controls and 13 AD patients) and/or from Actiwatch record analyses (4 controls and 4 AD patients) were used.

**Fig 2:**
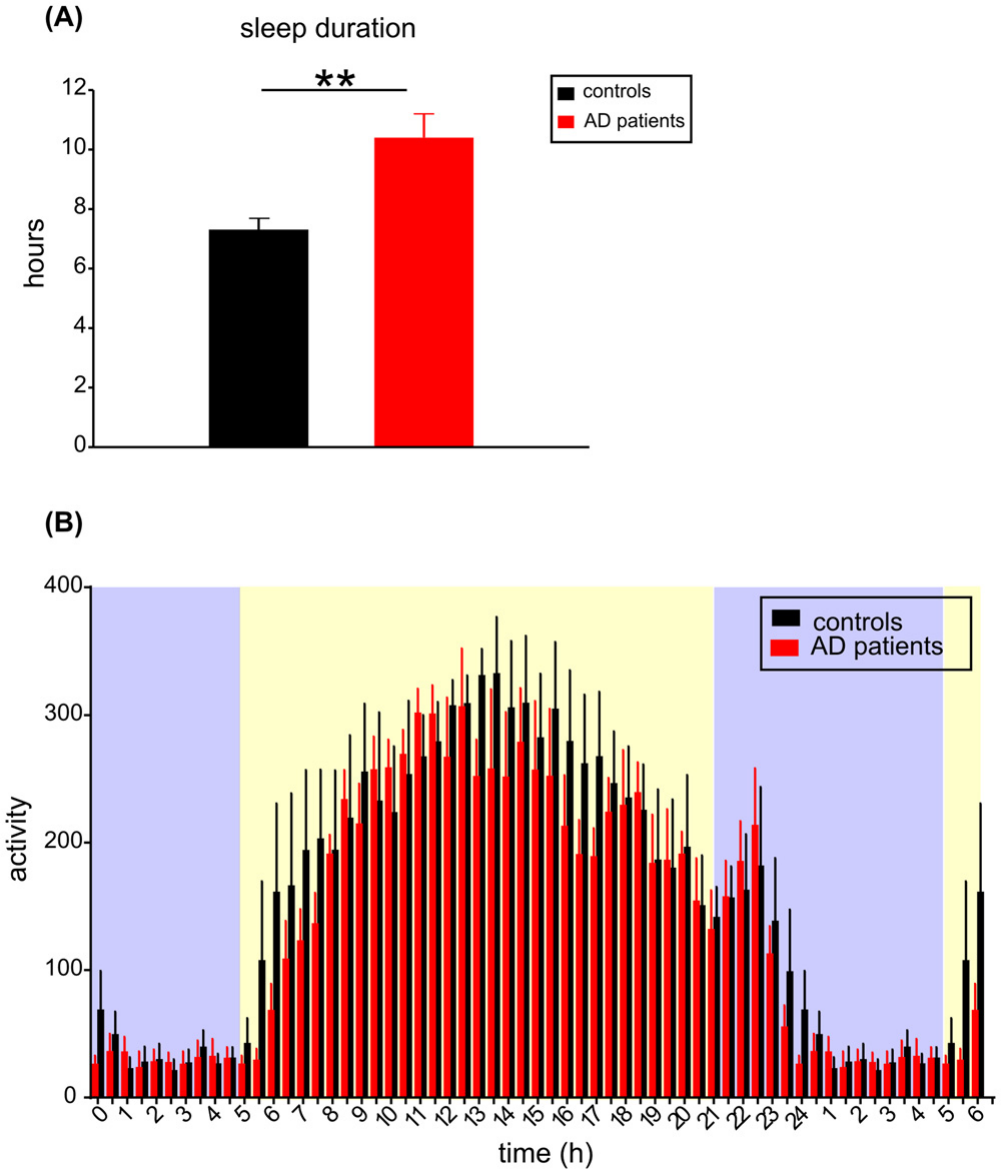
Analysis of sleep and activity rhythms of controls and AD patients. (A) Total sleep duration of the 24-h period (in hours) was assessed by the diary data (mean ± SEM) collected during the 21 day recording period from 13 controls (black column) and 13 AD patients (red column). In AD patients, the mean total sleep duration was significantly longer compared to controls, ** P = 0.002; (B) The mean daily activity profiles of AD patients and controls. The activity was recorded by Actiwatch during 21 days and the cumulated activity levels (mean ± SEM) in 30 min bins throughout the day and night are depicted; for clarity, part of the day (00:00 to 06:00 h) was re-plotted. The activity levels in 4 controls (black columns) and 4 AD patients (red columns) are depicted. The blue and yellow areas on the graph background correspond with time of the environmental darkness and daylight, respectively, as occurred during the recording period. X axis represents clock time (hours). For more details, see Material and Methods.

Sleep time during the 24-h period was analyzed in all participants of the study based on records in their sleep diaries. The total time of sleep duration during the 24-h period (Fig 2A), expressed as mean ± SEM of the 21 day recording period, was 7.3 ± 0.4 h in controls (n = 13) and 10.4 ± 0.8 h in AD patients (n = 13). In AD patients, the mean total sleep duration was significantly longer compared to controls (Student's t-test, P = 0.002). Additionally, daily activity/rest profiles cumulated for the 21 days of the recording period obtained from the Actiwatch records of 4 patient/control couples are depicted in Fig 2B. The repeated measures two-way ANOVA revealed significant effect of time (F = 17.020; P < 0.0001) confirming the presence of daily variation in activity for both groups; however, these activity/rest profiles did not differ significantly (F = 0.394; P = 0.553) between both groups.

**Fig 3:**
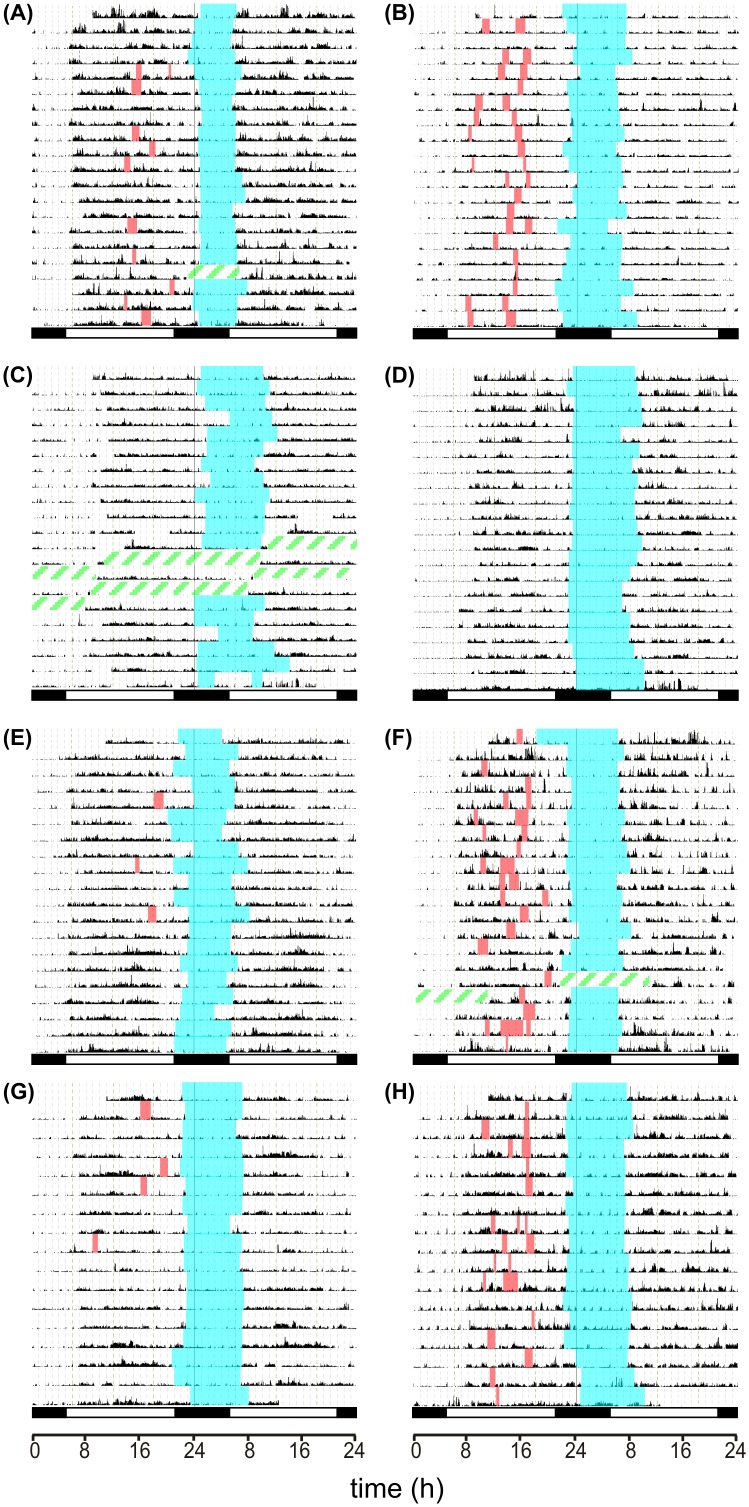
Individual actigraphs of the control/AD patient couples. Actigraphs from controls (A,C,E,G) and AD patients (B,D,F,H) are depicted, representing the AB, CD, EF and GH couples. The activity was recorded during 21 days (A-F) or 17 days (G,H). The record of activity (black area) from the Actiwatch analysis was completed with manual marking of nighttime sleep periods (blue area) and daytime naps (orange area) according to data from the individual sleep diaries. The green dashed areas represent intervals when the device was not operating.

The analysis of sleep parameters was performed by the Actiwatch software, and/or using records of the sleep diaries, separately for the night sleep and the daytime naps. The interval designated in the diaries by the subjects as time of falling asleep at night and waking up in the morning was considered the nighttime sleep and the intervals designated by the subjects as short sleep during the day were considered as naps. In one AD patient (AD#103; data not shown), the sleep was fragmented, occurring in regular intervals throughout the day and night and, therefore, the nighttime sleep and daytime naps could not be distinguished. The couple of the AD#103 patient/control was thus excluded from this part of analysis. For all other subjects, the nighttime sleep was more or less consistent and clearly distinguishable from the sporadic daytime napping. The actigraphs of 4 control/AD patient couples recorded throughout the 21-day period are depicted in Fig 3; the intervals designated in the sleep diaries as the nighttime sleep and the daytime naps are marked. One of the 4 couples reported not having naps (see below).

**Fig 4:**
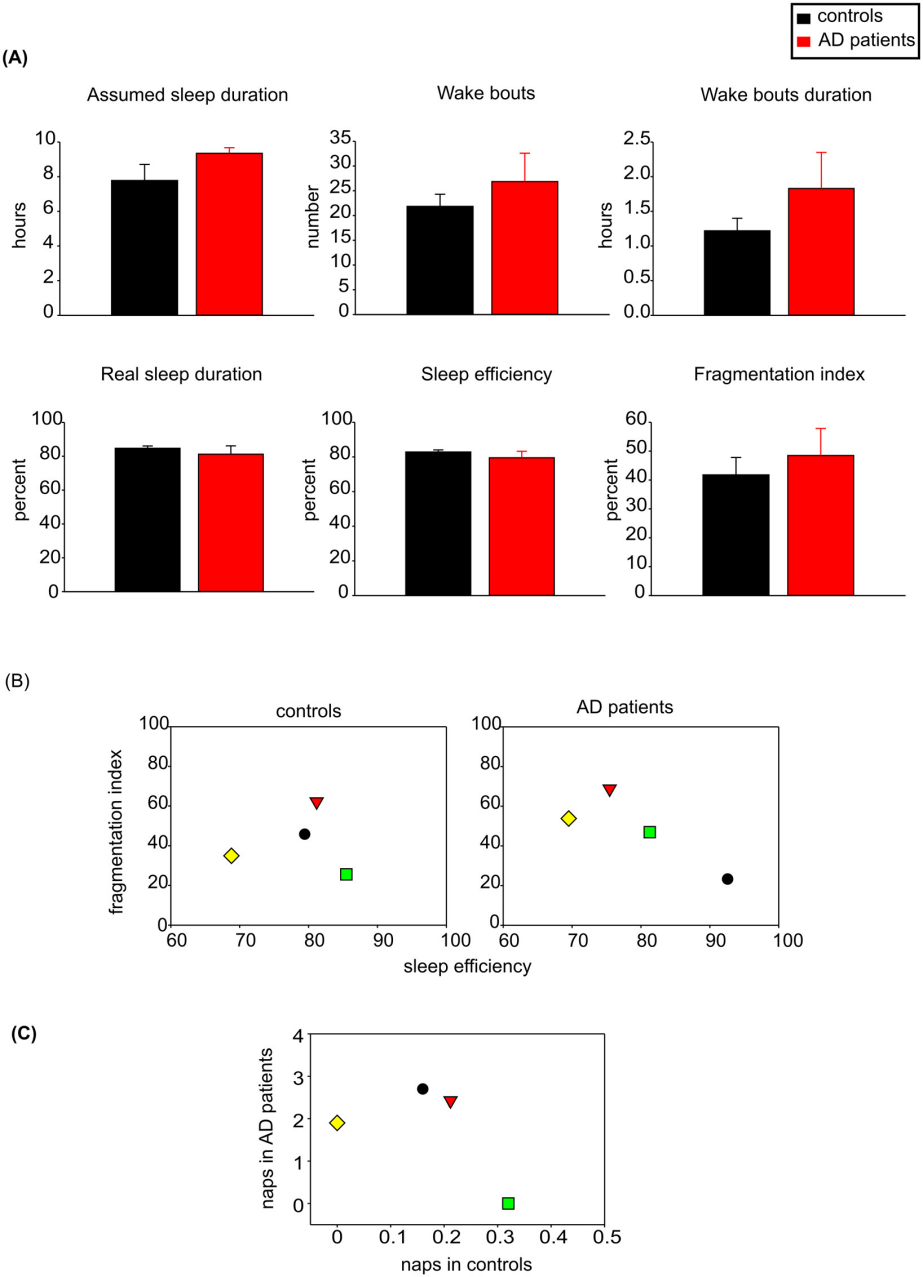
Nighttime sleep parameters and naps of controls and AD patients. (A) The nighttime sleep parameters (mean ± SEM), i.e., the length of the assumed sleep duration (hours), the number of awake episodes, the duration of awake episodes (hours), the real sleep duration (percent), the sleep efficiency (percent) and the fragmentation index (percent) were detected by Actiwatch analysis and compared between the group of 4 controls (black columns) and 4 AD patients (red columns); (B) The values of the sleep efficiencies and fragmentation indexes of the individual controls (left side) and AD patients (right side). For identification of the control/AD patients couples, the subjects of each couple are depicted with the same symbol (couple 1: yellow diamond, couple 2: black dot, couple 3: red triangle, couple 4: green square); (C) Mean number of daytime naps as detected by Actiwatch analysis (and confirmed by the sleep diaries records) for the control/AD patients couples. The symbols identifying the couples correspond to those depicting the nighttime sleep parameters in B). For more detail, see Material and Methods.

The mean assumed nighttime sleep duration determined from the sleep diaries was 7.3 ± 0.3 h (n = 12) in controls and 9.1 ± 0.7 h (n = 12) in AD patients. For the AD patient/control couples, whose sleep parameters were analyzed by the Actiwatch software, it was 7.8 ± 0.6 h (n = 4) in controls and 9.4 ± 0.4 h (n = 4) in AD patients. Although both measurements showed longer assumed sleep duration in AD patients than in controls, the differences between both groups were not statistically significant, be they detected by the diary records or Actiwatch software (Student's t-test; P = 0.056 and P = 0.071, respectively). The analysis of the sleep parameters by the Actiwatch software detected the number and duration of wake bouts, percentage of real sleep duration, sleep efficiency and fragmentation index (see Methods for definition). As demonstrated in Fig 4A, none of the sleep parameters were significantly different between both groups and there was only a trend in AD patients towards slightly longer assumed sleep duration (P = 0.071). The sleep efficiency and sleep fragmentation also did not differ significantly between both groups (P = 0.550 and P = 0.569).

The mean number of daytime naps during the 21-day recording period was counted for each subject based on the data from the sleep diaries. Most subjects of both groups reported napping; frequency of the naps was variable within and among all subjects and only one of the 13 AD patients and 5 of the 13 controls reported not having daytime naps throughout the 21-day period of recording. The mean number of daytime sleep bouts in AD patients was significantly higher (0.81 ± 0.23, n = 12) than in controls (0.25 ± 0.13. n = 12) (Student's t-test; P = 0.046). The same borderline significance was reached also for data acquired from Actiwatch analysis (Student's t-test; P = 0.041) where the mean number of daytime naps was significantly higher in AD patients (1.76 ± 0.61, n = 4) than in controls (0.17 ± 0.05, n = 4). Moreover, the Actiwatch analysis suggested that duration of naps might be longer in AD patients (0.62 ± 0.32 h, n = 4) than in controls (0.12 ± 0.02 h, n = 4), but the difference was not statistically significant (Student's t-test; P = 0.170).

The distribution of individual data for sleep parameters and daytime naps in each of the 4 control/AD patient couples is depicted in Fig 4B and 4C. The data revealed that in 3 out of the 4 couples, the patients had lower or the same sleep efficiency and higher fragmentation index compared with their corresponding control spouses. Nevertheless, even in controls the individual values of both markers demonstrated high variability. Interestingly, in one couple, the AD patient had a better nighttime sleep quality than the control (his sleep efficiency was higher and fragmentation index was lower); however, this one AD patient exhibited also the highest mean number of daytime naps compared with other AD patients who exhibited poorer sleep quality based on their sleep efficiency and fragmentation index (Fig 4C). Therefore, the better night sleep quality in this patient was not in parallel with less sleep during the daytime, it rather reflected higher total sleep time during the 24 h. In summary, from comparison of the individual sleep and nap parameters it appeared that in the control subject, the poor night sleep quality (sleep efficiency lower than 75%) was not compensated by higher daytime napping and that AD patients had more frequent daytime naps even in case of good night sleep quality.

### Mean daily profiles of melatonin levels are not significantly different but more individual profiles are aberrant in AD patients than in controls

**Fig 5:**
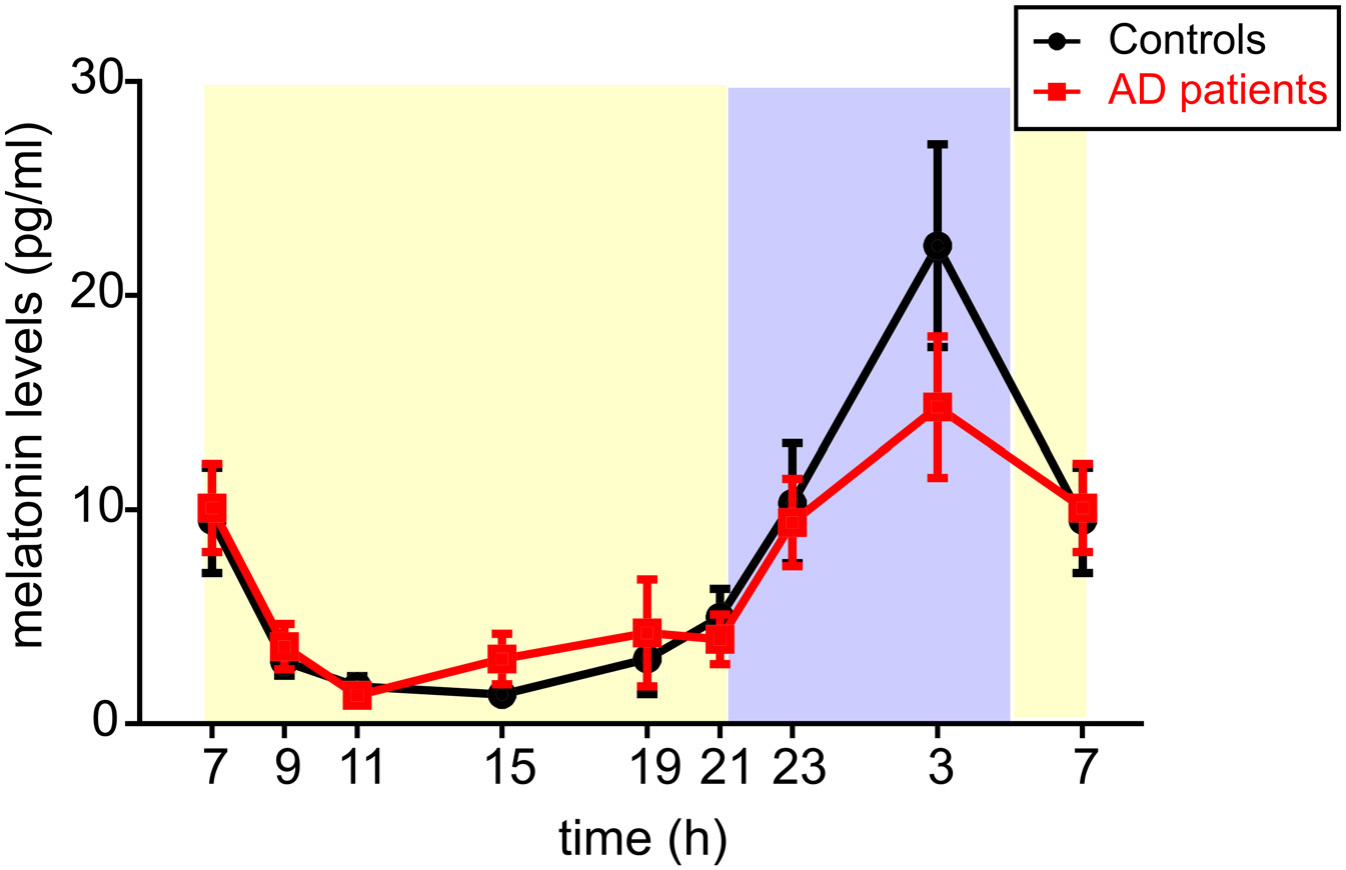
Daily profiles of melatonin levels in the saliva of controls and AD patients. Melatonin levels were detected during the 24 h and expressed in pg/ml. (A) The daily melatonin profiles expressed as the means ± S.E.M. in controls (black circles and black lines, n = 13) and AD patients (red squares and red lines, n = 13); (B) The individual melatonin levels in controls (black circles) and AD patients (red squares) are depicted. Moreover, the mean cosine fits (black curve for controls and red curve for AD patients) were obtained by the cosinor analysis. X-axes represent the time of day in hours, the blue and yellow areas on the graphs backgrounds correspond with time of the outside darkness and daylight, respectively, as occurred during the recording period.

In each subject, the melatonin levels were determined at 9 time points during the 24 h. The daily profiles of the mean melatonin levels for the groups of controls and AD patients (mean ± SEM, n = 13 for each group) are depicted in Fig 5. The repeated measures 2-way ANOVA revealed significant effect of time (F = 18.470; P < 0.0001); however, the difference between the mean melatonin profiles in AD patients and controls was not significant (F = 0.026; P = 0.874). The result was confirmed by calculation of area under the curve (AUC) for each profile. These values were averaged for each group and then compared between AD patients and controls by Student's t-test (P = 0.585).

Examination of individual melatonin profiles revealed a high variability within both groups (data summarized in Table 1). From the 13 control subjects, 8 subjects exhibited the typical rhythmic profiles (in 7 of them the amplitudes were between 20 and 40 pg/ml and in 1 of them it was 60 pg/ml). In 4 control subjects, the melatonin levels did not rise during the night (the nighttime levels were lower than 5 pg/ml) and the profile did not exhibit circadian rhythm. One control subject had an advanced low-amplitude rhythm (maximal levels of approximately 20 pg/ml at 21:00 and 23:00). From the group of 13 AD patients, 6 subjects had melatonin profiles that did not exhibit circadian rhythms (the levels were suppressed throughout the 24-h interval to less than 5 pg/ml), or the rhythm was shallow and amplitudes were very low (10 pg/ml or lower). In 4 AD patients, the melatonin profiles exhibited typical circadian rhythms with peaks at 03:00 and the amplitudes between 20–40 pg/ml. In 3 AD patients, the peak was advanced; in 1 case to 19:00 (20–30 pg/ml) and in 2 cases to 23:00 (10–20 pg/ml). From the analysis of individual profiles it appeared that the typical circadian rhythm with the peak during the nighttime was present more often in controls than in AD patients. Therefore, based on the analysis of the individual melatonin profiles, more aberrancies and lower amplitudes of the melatonin rhythms were present in AD patients compared to controls.

**Table 1:** Summary of the individual melatonin profile characteristics in controls and AD patients.

	Daily melatonin profile			
group	Total number	Circadian rhythms	Shallow rhythms	Atypical phase
Controls	13	8	4	1
AD	13	4	6	3

Number of daily melatonin profiles of controls and AD patients which exhibited circadian rhythm (nocturnal peak above 10 pg/ml), shallow rhythm (nocturnal peak below 10 pg/ml) or a rhythm with atypical phase. For detailed explanation, see Results.

### *PER1* and *BMAL1* daily expression profiles are not significantly affected in AD patients

**Fig 6:**
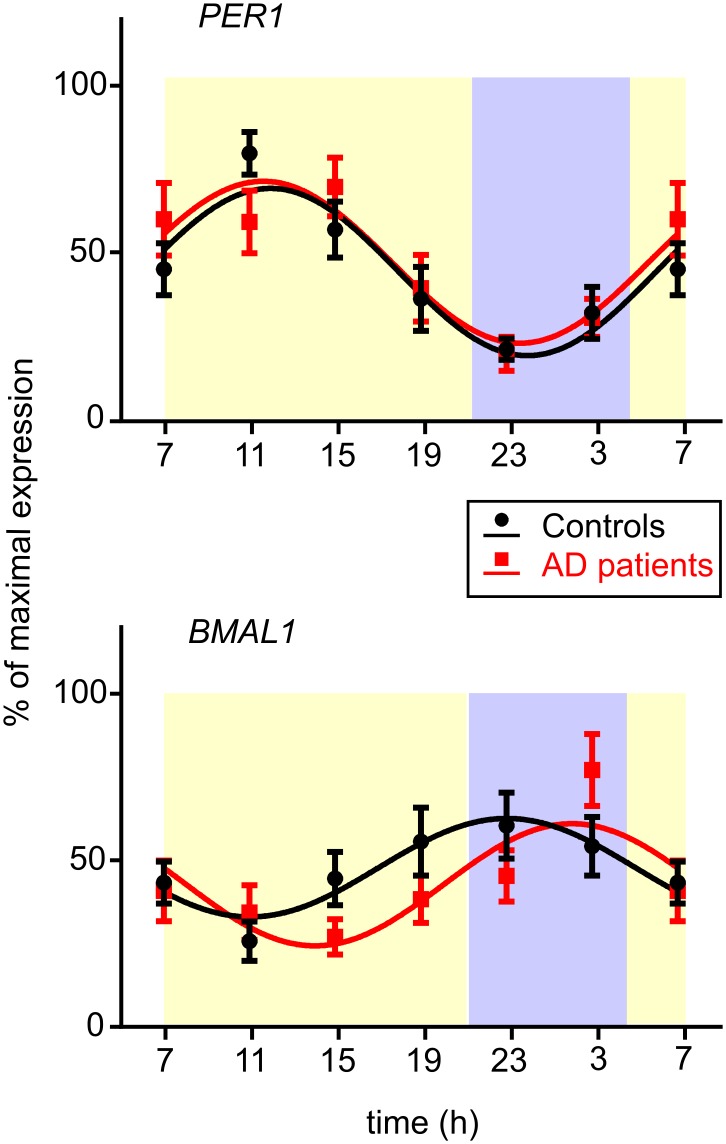
Daily profiles of clock gene expression in buccal cells of controls and AD patients. *PER1* (upper graph) and *BMAL1* (lower graph) expression profiles were compared in controls (black circles, n = 13) and AD patients (red squares, n = 13). Data are expressed as % of maximal expression levels (means ± S.E.M.) and fitted with cosine curves (controls: black curves, AD patients: red curves). The X-axes represent the time of day in hours.

The *PER1* and *BMAL1* expression profiles in buccal mucosa of controls and AD patients are depicted in Fig 6. The cosinor analysis of the daily profiles revealed significant circadian rhythms for both genes and experimental groups (Table 2). In controls and AD patients, *PER1* expression rhythms (Fig 6A) exhibited maximal levels (acrophases) during the day and the minimal levels during the night. Notably, *BMAL1* expression rhythm (Fig 6B) was in opposite phase to *PER1* in both groups. However, a closer inspection of phases of the clock gene expression rhythms suggested that whereas the *PER1* rhythm was expressed in the same phase in controls and AD patients, the *BMAL1* rhythm seemed phase-delayed in AD patients. The comparison of the expression rhythms between controls and AD patients using the repeated measures 2-way ANOVA revealed significant effect of time (*PER1*: F = 9.435, P < 0.0001; *BMAL1*: F = 4.637, P < 0.0003) but not significant difference between the clock gene expression profiles in AD patients and controls (*PER1*: F = 0.537, P = 0.471; *BMAL1*: F = 1.655, P = 0.211). Moreover, comparison of acrophases between both groups by unpaired Student's t-test did not reveal significant differences (*PER1*: P = 0.953; *BMAL1*: P = 0.278).

**Table 2:** Results of cosinor analysis of the clock gene expression profiles.

	PER1		BMAL1	
Cosinor analysis	Controls	AD patients	Controls	AD patients
**P**	< 0.001	< 0.001	0.0058	0.0015
**R**^**2**^	0.2831	0.2213	0.1105	0.1490
**Acrophase (mean ± SEM)**	12.0 ± 0.6	11.6 ± 0.7	23.0 ± 1.1	2.1 ± 0.9
**Amplitude (mean ± SEM)**	25.1 ± 4.3	24.3 ± 5.1	14.8 ± 4.5	18.3 ± 4.9
**Mesor (mean ± SEM)**	44.1 ± 2.9	47.1 ± 3.4	47.8 ± 3.1	42.7 ± 3.3

P (statistical significance); R^2^ (coefficient of determination); Acrophase (the phase angle of the peak of a cosine curve in hours); Amplitude (the difference between the peak or trough and the mean value of a cosine curve); Mesor (the average value around which the variable oscillates)

## Discussion

In this pilot field study, we examined actual state of the circadian system in patients with mild form of AD and healthy age-matched controls who lived together in their home dwellings and were thus exposed to the same environmental cues of their everyday life. Our results suggest that under real-life conditions, circadian regulation of behavior, melatonin levels and peripheral clock gene expression in the AD patients may modestly differ compared with the controls. The limitation of this study is a low number of enrollees (13 patients and 13 healthy controls); this was mainly caused by the special arrangement of the study. Due to the relatively high inter-individual variability of the data even in control elderly subjects, the low number of participants likely accounts for the fact that our results demonstrate trends rather than significant differences.

In AD patients, the circadian activity/rest rhythm was maintained, however, their inactivity interval during the 24 h assessed by sleep diaries was significantly longer compared with that in controls. The analysis of individual sleep/wake cycles revealed that the AD patients and controls exhibited more or less consistent rhythm in nighttime sleep and daytime wakefulness; only in 1 out of 13 patients the rhythm was completely abolished and the sleep occurred in bouts equally distributed over the 24 h period. Subsequent analysis of sleep parameters in 4 AD patient/control couples by Actiwach revealed that whereas the AD patients spent more time inactive in bed, their sleep was likely of poorer quality compared with controls. However, the worse sleep quality in AD patients was only suggested because differences in sleep efficiency and fragmentation index between both groups did not reach statistical significance. This might be related with the low number of subjects which we could include in the actigraphic study due to limited number of the devices. Additionally, the data revealed that the AD patients slept more often during the day as their number of daytime naps was significantly higher than that in controls, be it assessed by sleep diaries in all subjects or by Actiwatch in 4 couples. Analysis of individual data revealed that the napping was present in nearly all AD patients (in 12 out of 13 patients) but less often in controls (in 8 out of 13 controls). Interestingly, one AD patient exhibited a better nighttime sleep quality than all examined controls (sleep efficiency higher than 90% and fragmentation index only about 20%) but the same patient also had the highest count of daytime naps. This case result suggests that the higher number of naps in AD patients might be a consequence of sleep/wake cycle disruption rather than an aftereffect to compensate the poor nighttime sleep quality.

Our data demonstrating the longer inactivity during the 24 h in AD patients compared to controls in their home-dwellings are in agreement with previously published findings in hospitalized patients [44, 45]. With age, the percentage of slow wave sleep and REM (rapid eye movement) sleep decreased [46]. Previous studies also revealed that in AD patients the incidence of the sleep/wake cycle aberrancies increased [15, 47]. During the nocturnal sleep, they exhibited significant increase in number and duration of wake up bouts and more pronounced decrease of SWS [15]. Additionally, during the day, they exhibited longer duration and higher frequency of daytime naps and the effect was associated with more profound functional impairments [47]. Unfortunately, our field study did not allow us to follow sleep parameters by polysomnography. However, our analysis of the less accurate actigraphy, which cannot distinguish between a state of wakefulness and a movement without awaking during the sleep, also suggested that sleep quality was likely poorer in AD patients than in their healthy spouses serving as their caregivers and living together with them in the same home-dwelling. The similarities in aberrancies of the rest/activity and sleep/wake cycles found in hospitalized AD patients and our home-living patients provides confirmation that they were not due to a worse adaptation of the AD patients to the hospital conditions. The factor of home environment has also been considered in several previous studies in AD patients. Accordingly, disturbances in the activity/rest rhythm were found in moderately demented home-dwelling AD patients [48]. However, in another study, differences between the AD patients living at home and healthy controls were only minimal and more pronounced disturbances of activity/rest rhythms were only demonstrated in hospitalized versus at home living AD patients [45].

The mean melatonin profiles exhibited circadian rhythms both in AD patients and controls. Decrease in amplitude of the melatonin rhythm in AD patients compared to controls was not significant, however, inspection of the individual profiles suggested a higher incidence of the rhythm disruptions (absence, abnormal shift of the peak) in AD patients than in control subjects. Generally high inter-individual variability was found in both groups and, therefore, we cannot be sure that the indicated differences were not due to relatively small sample size employed in the study. Nevertheless, another study confirmed decrease in melatonin rhythm amplitude already in preclinical cognitively intact patients [49]. The studies of melatonin levels in cerebrospinal fluid in postmortem samples collected from AD patients in advanced stage of the disease demonstrated more distinct suppression of the nocturnal levels [23], however, their light exposure history was unclear.

In our study, we cannot exclude a possible masking effect of environmental cues, mainly the light, on the endogenous melatonin production, because light was controlled only during the sleep hours on the night when samples were collected. Therefore, although we could not confirm a statistically significant effect of the disease on melatonin levels under real-life conditions, we cannot completely rule out this effect on the endogenous mechanism producing melatonin rhythm. On the other hand, we can exclude the possibility that the differences in melatonin profiles demonstrated in this study in AD patients were due to their different light exposure because the protocol of the study was arranged to ensure that the AD patient/control couples were together throughout the study and were thus exposed to the same environmental cues. In accordance with our results, another hormonal marker controlled by the circadian clock, cortisol, exhibited a significant circadian rhythm in moderately demented home-dwelling AD patients whose activity/rest cycle was disrupted [48]. This result, together with our data, supports the concept that in the AD patients in mild stage of the disease, the endogenous timekeeping mechanism in the central clock is likely preserved but its outputs regulating sleep, behavior and rhythmic hormonal production might become affected. The concept is further supported by our finding of robust circadian oscillation in clock gene expression in oral mucosa cells, providing final evidence that the cellular molecular clock mechanism in our AD patients was intact. Similar conclusion was drawn based on the results of a post-mortem study of circadian clock gene expression profiles in various brain areas in AD patients [35].

In summary, our results suggest that compared with their caregivers of the same age and exposed to the same environmental cues, the AD patients exhibit differences in activity/rest cycles which are manifested as significantly higher duration of the inactivity during 24 h and more daytime naps. In AD patients, in spite of the significant disruption in activity/rest and sleep/wake cycles, only marginal disruption, if any, in regulation of melatonin and clock gene expression profiles were detected. Therefore, the endogenous mechanism generating the circadian rhythmic signal is likely preserved during the mild stage of the disease. However, it is necessary to mention that the healthy controls were the patients' spouses who took care of them likely around the clock which might contribute to undervaluation of the results. Nevertheless, we believe that the experimental design of this pilot field study, testing the subjects in real-life situation, has its obvious relevance because it may reveal the effect of AD on adaptability of the circadian system to environmental conditions.

## Supporting Information

S1 Table Clinical and biomarker characteristics of 13 patients with Alzheimer disease involved in the study. Clinical status of the patients was characterized using Mini-Mental State Examination (MMSE) and Functional Activities Questionnaire (FAQ) [39, 50]. Changes on brain magnetic resonance imaging (MRI) were evaluated using medial temporal lobe atrophy (MTA) score [51]. The evaluation of MRI scans was done by scoring the extent of periventricular hyperintensities (PVH) and deep white matter lesions (DWML) according to the Fazekas scale [52]. Absence of such features is classified „0“, caps or pencil-thin lining of PVH– 1, smooth halos– 2 and irregular PVH extending into the deep white matter– 3. DWML constituting only punctate foci score 1, beginning confluent foci are 2 and large confluent areas of DWML were evaluated as 3. Each side of the mediotemporal region on Neurogam—processed SPECT 3D images was assessed by simple in-house unpublished semiquantitative scale (0 –negative, 1 –borderline, 2 –positive). A combination of scores from both sides resulted in total brain score: 0 –negative, 1 –borderline, 2 –positive on one side, 3 –positive on both sides. Cut-off cerebrospinal fluid concentrations were established on well characterized samples measured in our lab, i.e., 334 pg/ml for total tau protein, 57 pg/ml for phospho-tau protein p181-tau and 448 pg/ml for beta-amyloid.
(DOCX)
