# Supplementary material for: Moderate Changes in the Circadian System of Alzheimer's Disease Patients Detected in Their Home Environment
Source: PLoS One. 2016 Jan 4;11(1):e0146200. doi: 10.1371/journal.pone.0146200 (PMC4701009; doi:10.1371/journal.pone.0146200)
Supplement: S1 Table — Clinical status of the patients was characterized using Mini-Mental State Examination (MMSE) and Functional Activities Questionnaire (FAQ) [39, 50]. Changes on brain magnetic resonance imaging (MRI) were evaluated using medial temporal lobe atrophy (MTA) score [51]. The evaluation of MRI scans was done by scoring the extent of periventricular hyperintensities (PVH) and deep white matter lesions (DWML) according to the Fazekas scale [52]. Absence of such features is classified „0“, caps or pencil-thin lining of PVH– 1, smooth halos– 2 and irregular PVH extending into the deep white matter– 3. DWML constituting only punctate foci score 1, beginning confluent foci are 2 and large confluent areas of DWML were evaluated as 3. Each side of the mediotemporal region on Neurogam—processed SPECT 3D images was assessed by simple in-house unpublished semiquantitative scale (0 –negative, 1 –borderline, 2 –positive). A combination of scores from both sides resulted in total brain score: 0 –negative, 1 –borderline, 2 –positive on one side, 3 –positive on both sides. Cut-off cerebrospinal fluid concentrations were established on well characterized samples measured in our lab, i.e., 334 pg/ml for total tau protein, 57 pg/ml for phospho-tau protein p181-tau and 448 pg/ml for beta-amyloid. (DOCX) [file pone.0146200.s001.docx]

**S1 Table. Clinical and biomarker characteristics of 13 patients with Alzheimer disease involved in the study**

|  |  |  |  |  |  |  | **Brain magnetic resonance imaging** | | | | **Brain SPECT** | **Cerebrospinal fluid** | | |
| --- | --- | --- | --- | --- | --- | --- | --- | --- | --- | --- | --- | --- | --- | --- |
| **AD patient No** | **Disease duration (years)** | **Age (years)** | **Sex** | **MMSE (0-30 points)** | **FAQ**  **(0-30 points)** | **Follow-up** | **MTA score dx (0-4) abnormal ≥ 2** | **MTA score sin (0-4) abnormal ≥ 2** | **PVH** | **DWML** | **SPECT MT hypoperfusion (0-3) abnormal ≥ 2** | **Total tau abnormal > 334 pg/l** | **Phosho-tau abnormal > 57 pg/l** | **Beta-amyloid abnormal < 448 pg/l** |
| 1 | 10 | 68 | f | 18 | 16 | institutionalization | **2** | **2** | 1 | 0 | 1 | **173** | **1186** | 519 |
| 2 | 5 | 77 | m | 26 | 7 | death unrelated to AD | 1 | 1 | 1 | 1 | **2** |  |  |  |
| 3 | 3 | 82 | m | 15 | 27 | institutionalization | **4** | **4** | 2 | 2 | **3** |  |  |  |
| 4 | 5 | 83 | f | 19 | 17 | progression to death | **4** | **3** | 1 | 1 | **3** |  |  |  |
| 5 | 3 | 79 | m | 25 | 12 | stable on anti-dementia drugs | **4** | **4** | 1 | 1 | **na** |  |  |  |
| 6 | 3 | 76 | f | 26 | 3 | stable on anti-dementia drugs | **2** | **3** | 3 | 3 | **3** | 225 | 43 | 1070 |
| 7 | 2 | 80 | m | 28 | 7 | progression | **2** | **2** | 0 | 0 | 1 |  |  |  |
| 8 | 3 | 78 | f | 12 | 18 | progression to death | **4** | **4** | 0 | 1 | **3** | 250 | 32 | 524 |
| 9 | 4 | 84 | f | 27 | 17 | mild progression | **3** | **4** | 2 | 2 | **3** |  |  |  |
| 10 | 2 | 82 | f | 22 | 15 | progression to death | KI | **KI** | KI | KI | **3** |  |  |  |
| 11 | 3 | 87 | m | 15 | 23 | institutionalization | **2** | **3** | 2 | 2 | **na** |  |  |  |
| 12 | 2 | 79 | m | 25 | 16 | progression to death | **3** | **3** | 3 | 3 | **3** | 290 | **68** | 302 |
| 13 | 3 | 63 | f | 20 | 24 | stable on anti-dementia drugs | 2 | **2** | 1 | 2 | na |  |  |  |

AD – Alzheimer disease, MMSE – Mini-Mental State Examination, FAQ – Functional Activities Questionnaire, MTA – mediotemporal atrophy, dx – on the right, sin – on the left, SPECT – single photon computed tomography, MT – mediotemporal hypoperfusion
